# Clinical pharmacy in Syria at a crossroads: challenges, opportunities, and pathways for post-conflict reform

**DOI:** 10.1007/s11096-026-02168-2

**Published:** 2026-05-29

**Authors:** Tarik Al-Diery, Ammar Al-Abdullah, Amani Zidan, Marah Mazen Jafar, Ayman Hameed, Salah Al-Deen Al-Safadi, Abdo Mahli

**Affiliations:** 1 College of Pharmacy, QU Health, Qatar University, Doha, Qatar; 2 Heart Hospital, Hamad Medical Corporation, Doha, Qatar; 3 Syrian Pharmacist Association, Damascus, Syrian Arab Republic; 4 Centre for Strategic Studies and Health Training, Damacus, Syrian Arab Republic; 5 Al-Sham Private University, Damascus, Syrian Arab Republic

**Keywords:** Clinical pharmacy, Curriculum design, Pharmacy education, Pharmacy residency, Syria

## Abstract

Following more than a decade of conflict and humanitarian crises, Syria’s health and higher education systems have experienced profound disruption, with lasting consequences for the pharmacy profession. Clinical pharmacy, in particular, remains underdeveloped despite its well-established potential to improve patient outcomes and support an overstretched healthcare system. This commentary critically examines the current landscape of clinical pharmacy education, training, and practice in Syria, highlighting the structural, regulatory, and cultural barriers that have constrained its advancement. Drawing on available evidence and the authors’ professional expertise in clinical pharmacy education and workforce development, the commentary examines the persistent theory–practice gap across undergraduate and postgraduate training and identifies key areas for reform. Building on this analysis, the commentary proposes context-sensitive future directions to strengthen clinical pharmacy in Syria. These include reforming competency-based curricula at the undergraduate and postgraduate levels, establishing hospital pharmacy residency programs accredited by pharmacy specific hospital organisations, investing in clinical pharmacy units within governmental hospitals, developing and supporting pharmacy preceptors and mentors, and fostering a culture of clinical and practice-based research. The role of professional pharmacy bodies and the Syrian diaspora in capacity building and mentorship is also emphasised as a bridge to achieving such desired outcomes. Ultimately, transforming clinical pharmacy in Syria will require coordinated leadership and sustained commitment from both the Ministry of Health and the Ministry of Higher Education. If aligned reform is pursued, clinical pharmacy can evolve from a marginalised practice into an essential pillar of Syria’s post-conflict health system, contributing to improved patient outcomes and long-term system resilience.

## The context of the Syrian civil war and its impact on the education and health systems

The Syrian uprisings that began in March 2011 and later escalated into a protracted civil war resulted in profound human loss and the systematic erosion of the country’s health and education systems [1]. In July 2022, the United Nations High Commissioner for Human Rights reported that at least 350,209 civilians were documented as killed between March 2011 and March 2021 [2]. This figure, however, is widely regarded as a conservative estimate, failing to capture undocumented deaths among combatants, deaths in detention, and the large-scale forced displacement of civilians [3]. Thousands were imprisoned, tortured, or killed extrajudicially, highlighting the scale of one of the gravest humanitarian crises since the Second World War.

In response to widespread human rights violations, international sanctions were imposed on the Syrian state, severely restricting financial flows, academic exchange, and institutional collaboration. These measures, alongside Bashar Al-Assad’s regime prioritisation of military control over public welfare, precipitated a marked decline in both healthcare delivery and higher education quality [1]. By late 2024, the cumulative strain of prolonged conflict, regional instability, and overextension of state resources enabled opposition forces to regain control of large parts of the country, culminating in the collapse of a 54-year authoritarian regime in December 2024.

Beyond the immediate violence, Syria’s one-party political system had long suppressed intellectual freedom and professional advancement, accelerating a sustained “brain drain” of academics, clinicians, and educators seeking safer and more enabling environments abroad [4]. During the period of active conflict (2011–2024), Syria’s once reputable higher education sector deteriorated rapidly. Targeted assassinations of senior academics, chronic underfunding, and salaries falling below $200 per month forced many faculty members to leave their posts [1].{Milton, 2019 #1} As a result, numerous undergraduate programs, particularly in public universities, were delivered with limited senior academic oversight, often relying solely on instructors with masters-level qualifications [1].

As Syria emerges from one of the darkest periods in its long and ancient history, a profound challenge—and opportunity—lies ahead: reimagining clinical pharmacy education, training, and practice to optimise patient health outcomes while responding to the growing demands placed on a fragile healthcare system. This transitional moment offers a rare opportunity to move beyond recovery toward purposeful reform, positioning clinical pharmacy as an integral component of patient-centred care in Syria [5].

This commentary aims to critically examine the current landscape of pharmacy education and practice in Syria, identify key structural gaps limiting the advancement of clinical pharmacy, and propose strategic, context-sensitive directions for future development. Drawing on existing evidence and the authors’ professional insights, this work seeks to contribute to the foundation of a sustainable and reputable clinical pharmacy system capable of supporting Syria’s evolving healthcare needs.

## Pharmacy education and training in Syria: a theory-practice gap

### Pharmacy education in Syria

Prior to the conflict, Syria had 19 Faculties of Pharmacy (six public and 13 private), all accredited by the Ministry of Higher Education, with postgraduate programs offered at Damascus, Aleppo, and Tishreen Universities [6]. The fragmentation of state authority during the war led to the emergence of new universities, particularly in northwestern regions, aimed at serving displaced populations [7]. Across both public and private institutions, pharmacy education follows a five-year, semester-based program culminating in a Bachelor of Pharmacy degree [6]. While several countries in the Middle East—including Lebanon, Jordan, Egypt, and nations within the Gulf Cooperation Council—have transitioned to a Doctor of Pharmacy (PharmD) as the terminal degree, a clinically focused degree remains absent within Syrian pharmacy education [8–10].

Undergraduate pharmacy curricula in Syria are predominantly theory-driven, with substantial emphasis placed on pharmaceutical sciences, analytical chemistry, and clinical biochemistry [6, 11]. This orientation reflects historical workforce demands linked to the expansion of Syria’s pharmaceutical manufacturing sector following partial economic reforms in the 1990s [6, 12]. As a result, clinical pharmacotherapy, although nominally embedded within curricula, remains underrepresented. Professional practice and skills-based courses are often delivered didactically, with limited use of simulation or authentic clinical scenarios. Interprofessional education is also largely absent, with health disciplines typically trained in isolation rather than through collaborative learning models.

### Experiential education

Experiential and workplace-based training is a cornerstone of pharmacist preparation in many high-income and developing countries [13]. In the United States, pharmacy students complete a structured series of experiential rotations across community, hospital, and industrial settings through Introductory Pharmacy Practice Experiences during their didactic training, followed by Advanced Pharmacy Practice Experiences in their final year [14]. In settings such as the United Kingdom [15], Australia [16], and New Zealand [17], graduates are granted provisional registration during a supervised internship year in a workplace-based setting (community or hospital), with progression to full registration contingent on demonstrating competence for autonomous practice and lifelong learning. In contrast, experiential training represents a major weakness within Syrian pharmacy education. Although final-year students are required to complete approximately eight hours per week at approved placement sites—including community, hospital, or industrial pharmacies—many of these settings lack the infrastructure, supervision, or educational support necessary for meaningful workplace-based learning [6]. Consequently, opportunities to develop clinical decision-making skills remain limited, a gap further exacerbated by the prolonged effects of conflict [18]. While placement sites are formally approved by university councils, enforcement has been inconsistent, with reports of inadequate student attendance and unethical sign-off practices during the war years.

### Postgraduate education

Postgraduate pharmacy training in Syria remains underdeveloped and largely confined to academic pathways [19]. Master’s and Doctor of Philosophy (PhD) programs are offered at Damascus, Aleppo, and Tishreen Universities, primarily within the pharmaceutical sciences [19]. The Ministry of Health also oversees a small number of pharmacy residency programs focused on laboratory diagnostics and pharmaceutical manufacturing and quality control [19]. To date, however, Syria lacks nationally or internationally accredited clinical pharmacy residency programs comparable to those established in countries such as the United States [20] and, more recently, Australia [21]. The absence of structured, clinically oriented residency training governed by a professional pharmacy body represents a significant barrier to advancing clinical pharmacy education and practice nationwide.

## Pharmacy practice in Syria: workforce distribution, career pathways, and the marginalisation of clinical roles

### Workforce distribution and the nature of pharmacy practice in Syria

The theory–practice gap observed in Syrian pharmacy education is mirrored—and reinforced—by the structure of pharmacy practice itself [12]. Pharmacists in Syria are predominantly employed in community and industrial settings, with hospital-based and clinically oriented roles occupying a comparatively marginal position within the workforce [6, 12]. This distribution has profoundly shaped professional identity, career aspirations, and the practical relevance of undergraduate training [12].

Career preferences among pharmacy graduates appear to reflect this practice landscape, driven largely by job availability, financial stability, and perceived professional security [12, 19]. Clinical roles, where available, are often viewed as ill-defined, unsupported, and professionally risky, offering limited opportunities for advancement or recognition [19]. A 2018 review of pharmacy education and practice reported that, at the time, Syria had 30,774 registered pharmacists, of whom approximately 17,841 were actively practicing and maintaining registration with the Syrian Pharmacist Association [6]. More than three-quarters of practicing pharmacists were employed in community or retail pharmacy settings, while a further 11.7% worked within the pharmaceutical industry. In contrast, fewer than 2% of pharmacists were engaged as pharmacy residents within Ministry of Health-affiliated programs. Although accurate workforce data following regime change remain unavailable, the overall distribution of pharmacists across practice sectors is unlikely to have shifted substantially; however, new data is required in the near future to better conceptualise the situational reality of pharmacy in Syria.

Clinical pharmacy practice in Syria remains significantly underrepresented, particularly in rural areas and conflict-affected provinces in the northwestern and eastern regions of the country. Where clinical pharmacy activities exist—most commonly in large government hospitals in Damascus—they are often informal and lack defined career pathways [5]. Pharmacists in these settings are frequently confined to medication distribution and supply-related roles rather than recognised as medication therapy experts. Although individual initiatives, such as participation in ward rounds or leading journal clubs, have been reported, these efforts reflect personal motivation rather than institutional expectation. Such roles lack formal job descriptions, credentialing processes, accountability frameworks, or mechanisms for continuing professional development and outcome evaluation. In this context, clinical pharmacy exists in fragmented and opportunistic forms rather than as an embedded component of healthcare delivery.

### Postgraduate education aspirations

The dominance of community and industrial pharmacy pathways also influences postgraduate education aspirations among Syrian pharmacy graduates [19]. A 2011 study involving 265 final-year students at Damascus University found that the most preferred areas for further study were biochemistry and laboratory diagnosis, followed by pharmaceutics and pharmaceutical industry, and then clinical pharmacy [19]. These preferences were shaped by perceived job availability, feasibility, and employment security.

Although clinical pharmacy was not formally established within the Syrian healthcare system prior to the 2011 uprisings, interest in the discipline was driven by its perceived scientific prestige and its association with direct patient care [19]. When examining postgraduate pathways, approximately 80% of respondents expressed preference for academic postgraduate programs, while only 17% indicated interest in residency programs managed by the Ministry of Health [19]. The limited appeal of residency training may reflect the absence of internationally recognised accreditation, unclear training frameworks, and the predominantly laboratory- and diagnostics-focused nature of existing residency programs. The lack of structured clinical residency pathways has therefore diminished the attractiveness of clinical pharmacy as a viable postgraduate trajectory [6].

### Cultural and structural tensions leading to the marginilisation of clinical pharmacy

Workforce patterns and postgraduate career preferences in Syria are not merely a consequence of individual choice but are structurally reinforced by regulatory, institutional, and cultural factors. Unlike healthcare systems where clinical pharmacy is supported by formal scopes of practice, credentialing mechanisms, and funded postgraduate residency programs, Syria lacks a coherent framework defining pharmacists’ clinical responsibilities. There is no recognised progression pathway from entry-level practice to advanced clinical roles, nor are there systems for privileging pharmacists to provide specialised clinical services [6, 12]. In the absence of such structures, healthcare institutions have limited incentive to invest in clinical pharmacy positions, perpetuating a cycle in which clinical roles remain peripheral rather than integral to care delivery.

Interprofessional dynamics further constrain the development of clinical pharmacy practice in Syria. The absence of formal multidisciplinary team structures and interprofessional education in undergraduate curricula has entrenched a hierarchical, physician-centred model of care in which clinical authority is concentrated primarily within the medical profession [22]. Within this structure, pharmacists are often perceived as dispensers or technical support personnel rather than as medication therapy experts with specialised training in pharmacotherapy, patient safety, and treatment optimisation. Limited physician exposure to pharmacist-led clinical services during undergraduate and postgraduate training has contributed to low awareness and appreciation of pharmacists’ competencies, reinforcing professional silos and role ambiguity. Consequently, opportunities for collaborative practice remain limited, and pharmacists’ clinical input is frequently underutilised or excluded from therapeutic decision-making. These dynamics not only marginalise pharmacists’ contributions but also represent a missed opportunity in a health system facing significant physician workload and burnout [23], where effective interprofessional collaboration could enhance patient care and system efficiency.

While structural and hierarchical challenges may currently exist within the physician-pharmacist relationship, pharmacists must also actively recognise and embody their role as medication experts if they expect other healthcare professionals to perceive them in this way. This requires not only confidence in their expertise, but also a willingness to challenge existing cultural and systemic barriers. In doing so, pharmacists can help reshape these dynamics and foster greater recognition of their value within the interprofessional team.

The prolonged effects of conflict have compounded these structural and hierarchical challenges. Widespread infrastructure destruction, severe budgetary constraints, and workforce shortages have prioritised service continuity over professional role expansion [22]. In this context, pharmacy practice has been normalised as logistics and supply-focused, reinforcing the perception of clinical pharmacy as a *luxury* rather than a necessity [5]. Repositioning clinical pharmacists as essential members of multidisciplinary healthcare teams will require a paradigm shift at the level of the Ministry of Health, particularly in a post-conflict environment where optimising existing human resources is critical to improving patient and system-level outcomes. A coordinated effort to increase awareness of pharmacists’ skills and capabilities—led by the Ministry of Health and supported by pharmacy organisations at university and national levels—is imperative to challenge misconceptions of pharmacists as medication suppliers and reframe them as clinical experts who can serve as essential pillars of the Syrian health system [24].

## Future directions: rebuilding and advancing clinical pharmacy in Syria

As Syria enters a critical phase of post-conflict recovery, rebuilding the health system presents not only immense challenges but also a rare opportunity for structural reform. Clinical pharmacy—long overlooked and at times marginalised in the Syrian health system—has immense potential to contribute to not only improving patient health outcomes, but also be a cost effective mechanism for the Ministry of Health, as well-trained clinical pharmacist are positioned to not reduce patient harm, but also innovate and advance the health systems [25, 26]. However, realising this potential will require deliberate, coordinated, and sustained action across all facets of education, regulation, workforce development, and professional culture. The following future directions outline pragmatic and context-sensitive strategies proposed by the authors based on their expertise in pharmacist training and development and curriculum development for establishing a reputable and sustainable clinical pharmacy system in Syria.

### Revamping undergraduate and postgraduate pharmacy curricula

Reforming pharmacy education represents the foundational step in advancing clinical pharmacy practice in Syria. Current undergraduate curricula remain heavily theory-driven, prioritising pharmaceutical sciences and laboratory-based disciplines with inadequate investment to clinical pharmacotherapy, professional skills training, and clinical research training. While this orientation has historically aligned with Syria’s workforce demand for pharmaceutical manufacturing, the recent removal of state sanctions on Syria and the move to a free-market economy with Syria at the crossroad of trade between Asia and Europe will likely reduce this contemporary need. Syrian pharmacists must be equipped with the knowledge, skills, and attitudes to address the evolving needs of Syria’s health system and be capable of life-long learning and adaptation to the expanding scope of practice.

While a PharmD curriculum could alleviate some of the existing gaps mentioned earlier, the key to meaningful change involves refocusing undergraduate curricula to competency-based education models that emphasise patient-centred care, problem-based learning, and interprofessional collaboration [27, 28]. Beyond the discipline of pharmacy, Syria’ education model remains heavily theory and memorisation driven. However, such a pedagogy fails to address contemporary demands of pharmacists capable of practicing autonomously whilst having strong critical-thinking capabilities and practicing evidence-based medicine as the foundation of their professional identity [29]. Greater curriculum focus needs to be placed on clinical pharmacotherapy and professional skills courses, supported by integrated case-based learning, simulation training, and early exposure to authentic clinical environments. While governmental and private institutions should be afforded a degree of autonomy to establish such competency-based programs, the Syrian Ministry of Higher Education must oversee these programs to ensure that they satisfy accreditation standards of the ministry in the absence of a council for the accreditation of pharmacy programs.

Clinical experiential training in Syria requires a major overhaul with a significant increase in the number of hours of clinical training per year. Similar to experiential education training programs in the United States, United Kingdom, Australia, and New Zealand, pharmacy students should be exposed to early clinical placements, with training in clinical sites increasing gradually each professional year [29]. An intern training year is not embedded in the Syrian pharmacy curricula; however, the current landscape of experiential training is not sufficient to prepare trainees for autonomous, collaborative practice. Therefore, it is imperative that experiential training is significantly increased across in the pharmacy curriculum, with a minimum number of supervised hours exceeding 1500 h, as is commonly observed in the New Zealand [30] and Australian intern training programs [31]. These supervised hours should be absorbed in their respective pharmacy curricula, with increased numbers of hours in clinical and ambulatory-based practice settings.

Postgraduate education similarly requires realignment. Existing postgraduate pathways are largely confined to academic or laboratory-focused disciplines, with limited opportunities for clinically oriented training [19]. Developing postgraduate clinical pharmacy programs—whether through Master’s or PhD degrees that are clinically and practice focuse—would provide a platform for more trainees to pursue this route. However, such programs need to be developed in alignment with national health priorities such as chronic disease management and antimicrobial stewardship to ensure that future graduates are capable of maximising their training whilst also benefiting the health sector. Establishing structured career pathways for postgraduate qualifications with financial and positional privileges in clinical settings could pave the way for more trainees to pursue clinical-based degrees and follow a trajectory of clinical practice.

Curricular reform cannot occur in isolation by the respective universities or The Ministry of Higher Education. Rather, it must be undertaken collaboratively, involving academic institutions, clinical educator experts, and healthcare stakeholders from the national government to ensure alignment with real-world practice. Without such an alignment, educational reform risks reproducing the same theory–practice gap it seeks to address.

### Establishing nationwide hospital pharmacy residency programs overseen by hospital-specific pharmacy organisations

The Syrian Ministry of Health-led residency programs, while representing a positive step toward structured postgraduate training, do not fully address the aspirations of pharmacy graduates seeking clinical roles nor the pressing clinical needs of a constrained and recovering health system. Current residency offerings remain largely laboratory- and diagnostics-focused, with limited emphasis on patient-facing clinical practice [19]. As such, Syria remains in need of accredited clinical pharmacy residency programs that are explicitly designed to prepare pharmacists for advanced clinical responsibility, autonomous practice, and integration within multidisciplinary care teams.

Internationally, clinical pharmacy residency programs are commonly overseen by professional hospital pharmacy organisations, such as the American Society of Health-System Pharmacists in the United States [20] and Advanced Pharmacy Australia [21]. These organisations play a central role in accrediting training sites, defining competency frameworks, supporting preceptor development, and ensuring consistency and quality across residency programs. While the Syrian Ministry of Health is well positioned to oversee overarching residency structures and workforce planning, the operational governance, accreditation, and professional development of pharmacy residencies would be more effectively delegated to a dedicated hospital pharmacy organisation.

Such an organisation would be responsible for establishing clear competency-based frameworks that define residents’ scope of practice, supervision requirements, and progression toward autonomous clinical responsibility. Formal recognition of residency qualifications would enhance professional credibility and create defined clinical career pathways, addressing the current absence of structured progression for pharmacists seeking advanced practice roles. Importantly, residency training would introduce graduated responsibility and supervised decision-making, addressing unsafe transitions from undergraduate training directly into unsupervised practice.

The necessity of clinical pharmacy residency training in Syria extends beyond immediate service provision. Residency programs have been shown internationally to play a critical role in developing future clinical educators, preceptors, and professional leaders [32]. Syria faces a substantial shortage of senior clinical pharmacists and trained educators capable of sustaining educational reform and practice advancement. Residency training therefore represents a strategic investment in capacity building, enabling the development of a cadre of pharmacists equipped to lead clinical services, mentor future trainees, and contribute to workforce resilience [33].

In a post-conflict health system characterised by workforce shortages, physician burnout, and increasing medication complexity [23], residency-trained clinical pharmacists can play a vital role in optimising pharmacotherapy, reducing medication-related harm, and supporting multidisciplinary care. Establishing nationally coordinated, hospital-based pharmacy residency programs is therefore not a luxury but a *necessity* for strengthening clinical care delivery and ensuring the sustainable growth of clinical pharmacy practice in Syria.

### Ministry of Health funding for clinical pharmacy units in governmental hospitals

In addition to embedding clinical pharmacy residency programs within Syria’s major urban hospitals, substantial government funding is imperative to ensure that the vision of clinical pharmacy across all provincial hospitals can be realised. While resistance to new expenditure is an expected challenge in financially constrained systems such as Syria’s, it is essential to reframe clinical pharmacy as a *core* component of healthcare delivery rather than a supplemental luxury. Residency-trained clinical pharmacists, when positioned within collaborative multidisciplinary teams, are equipped to assume advanced clinical responsibility, support therapeutic decision-making, and contribute meaningfully to patient safety and system efficiency [34]. In this context, investment in clinical pharmacy represents a low-risk, high-reward strategy with the potential to reduce avoidable medication-related harm and downstream healthcare costs [25].

The Ministry of Health should therefore commit to establishing funded clinical pharmacy departments within all 14 provincial capitals over the next five years, with phased expansion into secondary hospitals in smaller towns over the following decade. Such a strategy would provide a clear national roadmap for service development while maximising Syria’s existing pharmacy workforce. Importantly, these departments should be anchored by structured clinical pharmacy residency programs to ensure a sustainable pipeline of competent practitioners capable of delivering, leading, and expanding services. Without residency-trained pharmacists, the scale-up of clinical pharmacy risks remaining superficial, dependent on individual initiative rather than institutional capacity.

Funding for clinical pharmacy services must extend beyond staffing to include infrastructure, training, and protected clinical time. Core funded activities should encompass medication optimisation, participation in ward rounds, medication safety and stewardship services, therapeutic drug monitoring, and provision of drug information and education to name a few. Without dedicated funding for these functions, pharmacists risk being relegated to medication supply and administrative roles, undermining the intent of clinical pharmacy reform. Meaningful investment from the Ministry of Health is therefore essential to embed clinical pharmacy as an integral, effective, and sustainable pillar of Syria’s post-conflict health system. Given that the role and service structures of physicians are already well established within the Syrian healthcare system, this may serve as a practical starting point for developing a parallel framework for clinical pharmacy services. Such an approach could help align expectations and provide a familiar reference for policymakers, thereby supporting the Ministry in more clearly understanding both the nature of the services being proposed and their potential benefits for patients and the healthcare system as a whole.

Enhancing clinical pharmacy program can also be achieved by public–private sector partnerships [35]. The Ministry of Health could set standards, with private entities providing resources and expertise, capitalising on the private sector's role in managing significant facilities [36]. One example of such a partnership would be to mandate that hospitals with more than 100 beds—including those with ICU capacity—establish one- to two-year residency programs and accommodate up to 10 graduates per cycle. Similar models have previously been implemented in countries such as Uganda, and these experiences can serve as valuable references when developing such programs [37].

### Developing and supporting pharmacy preceptors and mentors

The success of high-quality pharmacy training is contingent on skilled and supported preceptors capable of developing and nurturing both undergraduate and postgraduate trainees within authentic workplace-based settings. In Syria, many pharmacists who supervise students or junior colleagues do so without formal training, recognition, or institutional support. This challenge is further compounded by the absence of a national pharmacy board responsible for defining scopes of practice and routinely reviewing pharmacists’ credentials through evidence of continuing professional development. Without such regulatory oversight, there is limited assurance that pharmacy preceptors possess the competencies required to effectively train, supervise, and assess learners in clinical environments.

Clinical supervision is inherently relational, relying on trust, credibility, and psychological safety between mentor and trainee [38]. For trainees to develop clinical competence and professional confidence, they must be afforded learning environments that support progressive autonomy while encouraging vulnerability, reflection, and help-seeking. Syria’s current training landscape lacks such a nurturing framework, with junior healthcare professionals often exposed to high expectations without adequate support, contributing to burnout and disengagement [23]. One strategy to address this gap is the structured development of pharmacy preceptors through formal training in supervision, feedback, assessment, and professional role modelling. Internationally, initiatives such as preceptor teaching certificate programs in the United States [39] and the “Teaching on the Run” program in Australia [40] have demonstrated positive outcomes in enhancing mentorship quality, while also providing formal recognition and, in some settings, incentives linked to promotion or remuneration.

Mentorship should therefore be formally recognised as a core professional responsibility rather than an informal or voluntary role [41]. In a health system recovering from prolonged disruption, effective mentorship can support professional identity formation, resilience, and workforce retention, particularly for early-career pharmacists navigating limited clinical pathways. Syria’s professional diaspora represents a valuable and underutilised resource in this regard. Structured exchange initiatives, observership opportunities in Gulf Cooperation Council countries or other advanced health systems, and remote or in-person mentorship by experienced Syrian clinical pharmacists abroad could meaningfully support local preceptor development. Leveraging such collaborations would strengthen training capacity while fostering professional solidarity and continuity across generations of Syrian pharmacists.

### Engraining a culture of clinical pharmacy and practice-based research

Sustainable advancement of clinical pharmacy requires more than structural reform; it demands a cultural shift toward inquiry, evaluation, and continuous improvement. Embedding a culture of clinical pharmacy and practice-based research is therefore essential. Yet, this area remains significantly underdeveloped in Syria [5]. A 2022 review identified only nine clinical pharmacy and practice-based research publications originating from Syria between 2009 and 2019, illuminating the limited scholarly output in this field [42]. Multiple factors have contributed to this gap, including prolonged humanitarian crises, restricted institutional funding, and limited access to research infrastructure during the war period [43].

While these structural constraints are important, the most critical barrier to advancing clinical pharmacy research in Syria is the absence of sustained research mentorship [43]. Syrian medical and pharmacy graduates have consistently demonstrated motivation and commitment to scholarly work [44–46]; however, the mass exodus of experienced academics and clinicians has severely depleted local mentorship capacity [1]. Outdated, theory-driven postgraduate research programs further compound this challenge, leaving early-career researchers without adequate guidance in study design, methodology, and dissemination [47]. As a result, only a small proportion of postgraduate research projects conducted by medical or pharmacy residents are ultimately published [43]. Although many international publishers have waived article processing charges for Syrian researchers [48], barriers persist in accessing methodological support, research networks, and mentorship-elements that are far more influential than publication fees in cultivating research competence and confidence.

To address these gaps, pharmacists in Syria must be equipped with foundational research literacy, quality improvement skills, and exposure to implementation science [49]. In the current context, encouraging small-scale, locally relevant projects—such as medication safety audits, service evaluations, pharmacovigilance initiatives, and public perception studies-offers a pragmatic pathway to generating context-specific evidence [46]. Importantly, research activity should be embedded within routine clinical practice rather than confined to academic settings [47], with emerging clinical pharmacy departments explicitly supporting and protecting time for such activities.

Syria’s professional diaspora of clinical pharmacists represents a valuable resource in this regard. Strategic partnerships between universities, hospitals, and professional bodies – facilitated by diaspora mentorship, collaborative supervision, and capacity-building initiatives – can strengthen research training and dissemination. Over time, fostering a culture of practice-based research will enable the development of clinical pharmacy services that are responsive to local needs and system realities, rather than reliant on imported or externally driven models.

## Conclusion

Emerging from the ashes of a prolonged humanitarian catastrophe and decades of authoritarian rule, Syria stands at a pivotal crossroads in the evolution of its clinical pharmacy profession. Regulatory, structural, and cultural forces have converged over time to marginalise clinical pharmacy and constrain its contribution to patient care. An outdated, predominantly theory-driven undergraduate and postgraduate education system, at times misaligned with contemporary healthcare demands, has further alienated clinical pharmacy from routine practice. Compounding this challenge is the absence of professional pharmacy bodies capable of overseeing training, credentialing, and ongoing development, leaving a vacuum in the preparation of competent clinical pharmacists equipped to meet the needs of an evolving health system.

Nevertheless, this moment of transition also presents an opportunity. Reimagining clinical pharmacy in Syria requires more than isolated reform; it demands coordinated, system-level action across education, regulation, workforce development, and professional culture. Strategic partnerships must be fostered among all stakeholders involved in healthcare education and delivery, including academic institutions, professional bodies, clinicians, and policymakers. In a country confronting profound health system strain, the meaningful integration of clinical pharmacists offers a pragmatic and underutilised avenue to enhance medication safety, optimise therapy, and support overstretched healthcare teams.

While leadership from the Syrian Ministry of Health and the Ministry of Higher Education is essential, the transformation of clinical pharmacy cannot be achieved through policy alone. Sustainable change will require deliberate collaboration, professional accountability, and a shared commitment to patient-centred care. If aligned reform is pursued with intent and foresight, clinical pharmacy can evolve from a marginalised practice into a vital pillar of Syria’s healthcare system, contributing not only to recovery, but to long-term resilience and renewal.

## Data Availability

No datasets were generated or analysed during the current study.
